# Predictors of hospital mortality in adult trauma patients receiving extracorporeal membrane oxygenation for advanced life support: a retrospective cohort study

**DOI:** 10.1186/s13049-018-0481-6

**Published:** 2018-02-08

**Authors:** Meng-Yu Wu, Pin-Li Chou, Tzu-I Wu, Pyng-Jing Lin

**Affiliations:** 1 Department of Cardiothoracic Surgery, Chang Gung Memorial Hospital and Chang Gung University, Taoyuan, Taiwan; 2 Department of Surgery, Chang Gung Memorial Hospital and Chang Gung University, Taoyuan, Taiwan; 3 Department of Obstetrics and Gynecology, Wan Fang Hospital, Taipei Medical University, Taipei, Taiwan; 4 Department of Obstetrics and Gynecology, School of Medicine, College of Medicine, Taipei Medical University, Taipei, Taiwan; 5 School of Traditional Chinese Medicine, Chang Gung University, Taoyuan, Taiwan; 6 Taoyuan, Taiwan, Republic of China

**Keywords:** Extracorporeal life support, Post-traumatic acute respiratory distress syndrome, Post-traumatic cardiac arrest, Bleeding shock

## Abstract

**Background:**

Using extracorporeal membrane oxygenation (ECMO) to provide advanced life support in adult trauma patients remains a controversial issue now. The study was aimed at identifying the independent predictors of hospital mortality in adult trauma patients receiving ECMO for advanced cardiopulmonary dysfunctions.

**Methods:**

This retrospective study enrolled 36 adult trauma patients receiving ECMO due to advanced shock or respiratory failure in a level I trauma center between August 2006 and October 2014. Variables collected for analysis were demographics, serum biomarkers, characteristics of trauma, injury severity score (ISS), damage-control interventions, indications of ECMO, and associated complications. The outcomes were hospital mortality and hemorrhage on ECMO. The multivariate logistic regression method was used to identify the independent prognostic predictors for the outcomes.

**Results:**

The medians of age and ISS were 36 (27–49) years and 29 (19–45). Twenty-three patients received damage-control interventions before ECMO. Among the 36 trauma patients, 14 received ECMO due to shock and 22 for respiratory failure. The complications of ECMO are major hemorrhages (*n* = 12), acute renal failure requiring hemodialysis (*n* = 10), and major brain events (*n* = 7). There were 15 patients died in hospital, and 9 of them were in the shock group.

**Conclusions:**

The severity of trauma and the type of cardiopulmonary dysfunction significantly affected the outcomes of ECMO used for sustaining patients with post-traumatic cardiopulmonary dysfunction. Hemorrhage on ECMO remained a concern while the device was required soon after trauma, although a heparin-minimized protocol was adopted.

**Trial registration:**

This study reported a health care intervention on human participants and was retrospectively registered. The Chang Gung Medical Foundation Institutional Review Board approved the study (no. 201601610B0) on December 12, 2016. All of the data were extracted from December 14, 2016, to March 31, 2017.

## Background

Trauma is one of the leading causes of death among adults around the world [1]. The most common causes of early death in trauma patients are hemorrhagic shock, cardiopulmonary dysfunction, and severe brain damage. Therefore, controlling active hemorrhages, maintaining arterial oxygenation, and draining pneumothorax or cardiac tamponade are essential techniques in emergency departments to decrease the early mortality rate in trauma patients [2, 3]. For selected trauma patients who are in advanced shock or respiratory failure, extracorporeal membrane oxygenation (ECMO) can be a salvage therapy that bridges these young and previously healthy patients to recovery [4–9]. ECMO is a simplified version of heart-lung machine that can replace the cardiopulmonary function temporarily; it simply sucks in deoxygenated blood from the patient’s venous system and pumps oxygenated blood back to the patient [10]. Depending on the destination of the oxygenated blood, ECMO offers two operating configurations. Venoarterial (VA) ECMO bypasses the lungs and returns the oxygenated blood back to the aorta. Therefore, VA-ECMO can raise both the arterial pressure and arterial oxygenation. Instead of bypassing the lungs, venovenous (VV) ECMO returns the oxygenated blood back to the right atrium. This arrangement makes the venous blood to be refreshed by ECMO first then by the native lungs. Therefore, VV-ECMO provides a purely respiratory support without significant disturbances to the cardiopulmonary system. According to a recently published meta-analysis that enrolls 215 patients with ECMO-treated post-traumatic respiratory failure, ECMO shows a rescue rate of 50 to 79% and is considered a useful salvage treatment for post-traumatic respiratory failure [11]. Now ECMO has also become a new attempt to rescue patients with post-traumatic shock and some encouraging experiences are sporadically reported [4, 7, 9]. However, as an extracorporeal circulation, ECMO requires heparinization and thus carries a risk of hemorrhage [10]. According to the Extracorporeal Life Support Organization Registry International Report 2016 [12], the overall incidence of hemorrhage in ECMO-treated adult patients is around 34% (respiratory support) to 43% (cardiac support). The risk of intracranial hemorrhage is around 2–4% in this report. However, the risk of hemorrhage on ECMO should increase while applying this heparin-equipped therapy to patients with a significant trauma-induced coagulopathy (TIC) [13, 14]. The study was aimed at presenting our experience on ECMO used for post-traumatic cardiopulmonary dysfunctions and identifying the independent predictors of hospital mortality.

## Methods

### Study population

From August 2006 to October 2014, a total of 638 patients received ECMO for hemodynamic (venoarterial mode; *n* = 489) or pulmonary (VV mode; *n* = 159) support at Chang Gung Memorial Hospital. Among the 638 patients, 36 adults (age ≥ 20 years) were enrolled in this retrospective study due to post-traumatic shock or post-traumatic respiratory failure. Before the administration of ECMO, all of the patients had received a whole body CT to recognize the major bleeding sites and received essential procedures (surgery or transarterial embolization) to stop active bleeding. After the major bleeding sites were controlled, ECMO would be used to sustain the hemodynamics or ventilation in selected patients with profound shock (systolic arterial pressure < 60 mmHg), severe hypoxemia [PaO_2_/ FiO_2_ ratio < 70 mmHg under maximal mechanical ventilation (MV)], or in-hospital cardiac arrest (CA). The institutional review board of our hospital approved the protocol (CGMF IRB no. 201601610B0) and waived the necessity of individual patient consent.

### Managements of ECMO

The details of the principles and equipment in our practice of ECMO have been described in previous reports [8, 9, 15, 16]. According to the candidate’s need, the femoral-femoral VA-ECMO (cut-down cannulation) or the femoral-jugular VV-ECMO (percutaneous cannulation) are provided promptly. The ECMO circuit is heparin-bonded and primed with heparin-contained (2500 U/L) normal saline. To balance the thrombotic and hemorrhagic risks on ECMO, two anticoagulant strategies of ECMO are prepared. The heparin- titrated strategy includes a pre-cannulation bolus dose (5000 unit) and a continuous intravenous maintenance dose of heparin to keep a prolonged aPTT (45–55 s) during ECMO. This strategy is applied to patients who develop a need of ECMO after couple days of trauma, since their risk of hemorrhage on ECMO should be similar to general population. In the other hand, the heparin- minimized strategy waives the pre-cannulation dose and delivers the continuous maintenance dose of heparin after 48 h of ECMO. Approaching a near-normal aPTT (< 40 s) is the therapeutic goal in this period to achieve a satisfactory hemostasis. This strategy is applied to the patients who develop a need of ECMO soon after trauma or damage control interventions, since their risk of hemorrhage on ECMO should be increased due to the effects of Trauma-induced coagulopathy (TIC) [14]. During ECMO, the dose of vasopressors and the intensity of MV are gradually down-graded to maintain a normal mean arterial pressure (70–90 mmHg) and arterial oxygenation (PaO_2_ > 60 mmHg, PaCO_2_ 30-50 mmHg, and SaO_2_ > 90%). Continuous renal replacement therapy (CRRT) is used to maintain negative fluid balance in patients with a temporary renal failure. While signs of recovery are presented, the support of ECMO is tapered and stopped if possible. For patients hemorrhaging on ECMO, withholding heparin plus blood transfusion (RBC: plasma: platelet about 1:1:3) is often the first step to achieve hemostasis on ECMO. Endoscopic, angiographic or surgical hemostasis was launched with low threshold once the conservative treatment failed or is impossible to stabilize the hemorrhage. Protamine or antifibrinolytic agents would be provided to patients in whom an obvious source of hemorrhage is not found after a series of investigations.

### Data collection and outcome measures

We retrospectively reviewed the electronic medical records in each patient and collected their important demographic and clinical data before and during the administration of ECMO. The following variables were collected: age, gender, characteristics of trauma, injury severity score, damage control interventions,, duration of MV before ECMO, MV settings (peak inspiratory pressure, mean airway pressure, positive end-expiratory pressure, F_i_O_2_), duration of emergency room (ER) admission to ECMO administration, cardiac arrest and requiring ECMO-assisted cardiopulmonary resuscitation (E-CPR), the latest results of blood tests (arterial blood gas sampling, blood cell counts, creatinine, and total bilirubin) before ECMO, aPTT values before ECMO and during the first, 12th, 24th and 48th hour of ECMO, durations of hospital and ECMO stay, complications (hemorrhage, requiring CRRT, and brain events) of ECMO, and the outcome (survived or death in hospital). The brain events on ECMO included brain hemorrhage and brain infarction. The primary endpoint of this study was death in hospital, and the secondary endpoint was hemorrhage during ECMO.

### Statistical analysis

Statistical analyses were performed using SPSS for Windows (Version 15.0, SPSS, Inc., IL, USA). Because the dataset was small, the Mann–Whitney *U* test was used to conduct univariate comparisons of the independent variables. The Chi-square or Fisher’s exact test was used to compare the categorical variables. The level of statistical significance was set at *p* <  0 .05. Continuous variables with a *p* <  0.05 were dichotomized according to the cut-off values. The cut-off values were determined by the receiver operating characteristic curve (ROC) analysis. These dichotomized risk factors were tested by the multivariate logistic regression analysis with backward stepwise selection to identify independent predictors of hospital mortality and hemorrhage on ECMO.

## Results

### Univariate comparison

**Fig. 1:**
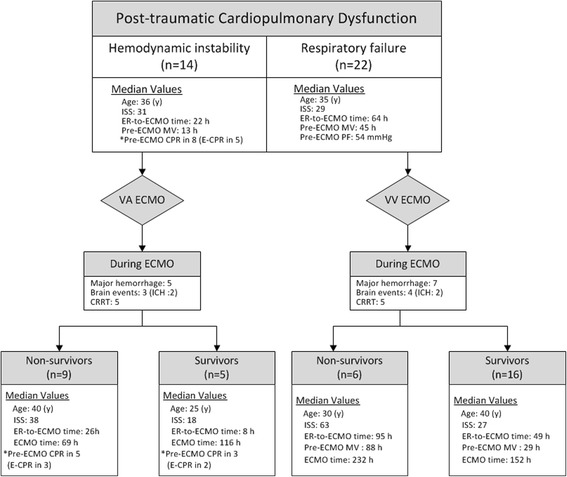
The flowchart of case distribution. CRRT: Continuous renal replacement therapy. ECMO: Extracorporeal membrane oxygenation. ER: Emergency room. ISS: Injury severity score. MV: Mechanical ventilation.VA: Venoarterial. VV: Venovenous

**Fig. 2:**
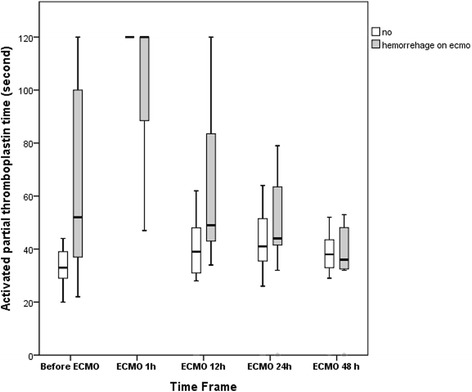
The time courses of the activated partial thromboplastin time during the first 48 h of extracorporeal membrane oxygenation. Median with 25th to 75th interquatile range. * The upper limit of detectable value of aPTT is 120 s

The median age and median ISS of the 36 patients were 36 (27–49) years and 29 (19–45), respectively. Among the 36 patients, 14 received VA-ECMO due to post-traumatic shock and 22 received VV-ECMO due to post-traumatic respiratory failure. In the group of VA-ECMO, eight patients required conventional CPR due to CA before ECMO institution. Three of the 8 patients regained spontaneous circulation after conventional CPR but still received VA-ECMO for the fluctuated hemodynamics. The other 5 patients developed refractory CA and eventually required E-CPR to restore spontaneous circulation. The median ECPR time was 35 (18–55). The causes of CA in the E-CPR group were severe airway obstruction with difficult intubation (*n* = 2), insufficient drainage of tension pneumothoraces/pneumomediastinum (*n* = 1), profound acidemia due to exsanguination during damage control surgery (*n* = 1), and profound sepsis due to intra-abdominal infection (*n* = 1). The patient with insufficient drainage of tension pneumothoraces/pneumomediastinum suffered from blunt chest injury with severe chest wall deformities, bilateral lung contusions, and pneumothoraces. He was treated with MV and bilateral tube thoracostomies initially and prepared for VV-ECMO implantation later due to his clinical deterioration. Unfortunately, he developed CA before the ECMO team arrived and he could just receive E-CPR with VA-ECMO. Twenty-four patients were weaned off ECMO after a median support of 130 (69–249) hours, and 21 of these patients survived to hospital discharge, including the 2 patients undergoing E-CPR. Figure 1 summarizes the clinical features among patients categorized by the need for ECMO (shock or respiratory failure) and the outcome (died or survived). The information on the mechanisms of trauma and associated therapies in each patient is summarized in Appendices 1 and 2. Twelve patients experienced major hemorrhage (4 intracranial, 5 intra-thoracic, and 3 intra-abdominal) on ECMO. Three patients were found to have significant brain infarctions. Ten patients experienced acute renal failure on ECMO and required CRRT. Table 1 lists the results of univariate comparisons between the survivors and non-survivors. Compared to the survivors, the non- survivors tended to have a higher ISS, post-traumatic shock/CA rather than respiratory failure, and a shorter hospital stay. In the VA-ECMO group, patients requiring E-CPR showed a similar hospital mortality rate when compared with patients receiving VA-ECMO for refractory shock(60% vs.67%; *p* = 1.0). On the other hand, the only significant difference between the hemorrhagic and the non-hemorrhagic groups was the value of aPTT obtained before ECMO (median aPTT: 52 vs. 33 s; *p* = 0.01). Table 2 demonstrates the results of univariate comparisons between the patients developing major hemorrhages on ECMO and the patients not. Compared to the non-hemorrhagic patients, the hemorrhagic patients tended to have a longer aPTT before ECMO institution. Figure 2 shows the trends of aPTT on ECMO in the hemorrhagic and non-hemorrhagic groups.

**Table 1:** Demographic and clinical data between survivors and non-survivors

	Survivor (*n* = 21)	Non-survivor (*n* = 15)	*P*
Pre-ECMO demographic data			
Age (year)	37 (26–51)	35 (27–47)	0.85
Male	19 (90)	12 (80)	0.63
ISS	25 (17–38)	41 (25–75)	0.03*
Traumatic brain injury	1 (5)	3 (20)	0.29
MV hours	14 (6–126)	26 (9–50)	0.87
Damage control intervention	13 (62)	10 (67)	0.77
ER-to-ECMO hours	21 (9–149)	37 (14–52)	0.75
Data obtained just before ECMO			
pH	7.29 (7.10–7.40)	7.20 (6.91–7.32)	0.14
Mean arterial pressure (mmHg)^a^	71 (53–92)	50 (44–59)	0.01*
PaO_2_ (mmHg)	53 (43–67)	47 (40–70)	0.55
PaCO_2_ (mmHg)	45 (39–70)	52 (46–87)	0.19
PaO_2_/FiO_2_ (mmHg)	54 (46–67)	49 (40–83)	070
Peak inspiratory pressure (cmH_2_O)	35 (31–38)	36 (32–48)	0.21
PEEP (cmH_2_O)	20 (15–24)	21 (15–24)	0.88
Hemoglobin (g/dL)	11 (9–14)	13 (9–15)	0.57
Platelet count (× 10^9^/L)	178 (90–199)	94 (69–190)	0.28
aPTT (second)	32 (28–41)	40 (35–62)	0.04*
Venovenous ECMO	16 (76)	6 (40)	0.03*
ECMO-assisted CPR	2 (10)	3 (20)	0.63
Complication on ECMO			
Major bleeding	4 (19)	8 (53)	0.07
Requiring CRRT	3 (14)	7 (47)	0.06
Brain event	4 (19)	3 (20)	1.0
ECMO hour	143 (76–219)	111 (36–297)	0.47
Hospital day	44 (29–78)	10 (5–23)	< 0.001*

Numerical variables are presented as median and interquartile range (IQR). Categorical variables are presented as number (percentage)

*aPTT* Activated partial thromboplastin time, *CPR* Cardiopulmonary resuscitation, *CRRT* Continuous renal replacement therapy, *ECMO* Extracorporeal membrane oxygenation, *ER* Emergency room, *ISS* Injury severity score, *MV hour* Mechanical ventilation hours before ECMO, *PEEP* Positive end-expiratory pressure, *Brain event* Brain hemorrhage or infarction

^a^Exclude patients with refractory cardiac arrest and requiring ECMO-assisted CPR

*: *p* <  0.05

**Table 2:** Demographic and clinical data between the patients developing major hemorrhages on extracorporeal membrane oxygenation and the patients not

	Hemorrhagic patient (*n* = 12)	Non-hemorrhagic patient (*n* = 24)	*P*
Pre-ECMO demographic data			
Age (year)	34 (25–52)	37 (27–49)	0.86
Male	9 (75)	22 (92)	0.31
ISS	33 (19–75)	29 (19–43)	0.54
Traumatic brain injury	2 (17)	2 (8)	0.59
MV hours	15 (5–44)	33 (8–139)	0.28
Damage control intervention	9 (75)	14 (58)	0.47
ER-to-ECMO hours	17 (6–42)	48 (12–166)	0.053
Data obtained just before ECMO			
pH	7.26 (7.13–7.34)	7.20 (7.09–7.37)	0.80
Mean arterial pressure (mmHg)^a^	54 (35–66)	64 (50–86)	0.16
PaO_2_ (mmHg)	48 (42–66)	52 (40–68)	0.88
PaCO_2_ (mmHg)	50 (39–70)	52 (39–72)	0.78
PaO_2_/FiO_2_(mmHg)	48 (42–66)	49 (40–83)	0.61
Peak inspiratory pressure (cmH_2_O)	35 (32–40)	35 (31–39)	0.88
PEEP (cmH_2_O)	22 (14–25)	19 (15–24)	0.75
Hemoglobin (g/dL)	12 (10–15)	11 (9–15)	0.61
Platelet count (× 10^9^/L)	93 (73–164)	171 (89–220)	0.07
aPTT (second)	52 (36–100)	33 (29–39)	0.01*
Venovenous ECMO	7 (58)	15 (63)	1.0
ECMO-assisted CPR	2 (17)	3 (13)	1.0
Complication on ECMO			
Requiring CRRT	4 (33)	6 (25)	0.70
Brain event	2 (17)	5 (21)	0.03*
ECMO hour	101 (45–248)	152 (71–271)	0.40
Hospital day	15 (6–50)	40 (24–51)	0.21

Numerical variables are presented as median and interquartile range (IQR). Categorical variables are presented as number (percentage)

*aPTT* Activated partial thromboplastin time, *CPR* Cardiopulmonary resuscitation, *CRRT* Continuous renal replacement therapy, *ECMO* Extracorporeal membrane oxygenation, *ER* Emergency room, *ISS* Injury severity score, *MV hour* Mechanical ventilation hours before ECMO, *PEEP* Positive end-expiratory pressure, *Brain event* Brain hemorrhage or infarction

^a^Exclude patients with refractory cardiac arrest and requiring ECMO-assisted CPR

*: *p* <  0.05

### Multivariate comparison

Before entering the multivariate test, the ISS was dichotomized at the point of 35 according to the results of ROC analysis. The sensitivity (Sn), specificity (Sp), positive predictive value (PPV), and negative predictive value (NPV) at this cut-off point for predicting hospital mortality were 67%, 76%, 67%, and 76%, respectively. With the same processing, the aPTT obtained before ECMO was also dichotomized at the point of 40. The Sn, Sp, PPV, and NPV at this cut-off point for predicting hemorrhage on ECMO were 67%, 67%, 59%, and 74%, respectively. Finally, an ISS > 35 and the requirement of VA-ECMO due to a refractory post-traumatic shock/CA were identified to be the independent predictors of hospital mortality of the ECMO-treated trauma patients. With the same method, a pre-ECMO value of aPTT > 40 s was proved to be the independent predictor of hemorrhage on ECMO. The indicators of diagnostic accuracy at this cutoff point were 67% (Sn), 79% (Sp), 62% (PPV), and 83% (NPV), respectively. Table 3 demonstrates the results of multivariate analysis. Table 4 presents the observed and predicted hospital mortalities calculated by the multivariate logistic regression model.

**Table 3:** Results of multivariate analysis

Endpoint	Predictor	β coefficient	Odds Ratio (95% CI)	*p* value
^a^Hospital Death	ISS > 35	1.905	6.716 (1.359–33.187)	0.019
	Shock/Cardiac arrest	1.625	5.079 (1.012–25.499)	0.048
^b^Hemorrhage on ECMO	Pre-ECMO aPTT > 40 s	2.028	7.6 (1.609–35. 906)	0.01

^a^Predicted mortality (y) = ℮^X^ / (1 + ℮^X^). X = − 1.853 + 1.905 × (Injury severity score > 35) + 1.625 × (post-traumatic shock or cardiac arrest). Hosmer-Lemeshow test *p* = 0.985; Negelkerke R^2^ = 0.354; c-index = 0.79

^b^Predicted risk of hemorrhage on ECMO (y) = ℮^X^ / (1 + ℮^X^). X = − 1.558 + 2.028 × (Pre-ECMO aPTT > 40 s)

Negelkerke R^2^ = 0.253; c-index = 0.73

**Table 4:** The observed and predicted hospital mortalities in the patient cohort

	Class I	Class II	Class III	Class IV
Indication of ECMO	Respiratory failure (ISS ≤ 35)	Shock/CA (ISS ≤ 35)	Respiratory failure (ISS > 35)	Shock/CA (ISS > 35)
Total Number	14	7	8	7
Death Number	2	3	4	6
Observed Mortality	14.3%	42.9%	50%	85.7%
Predicted Mortality^a^	13.5%	44.3%	51.3%	84.2%

^a^Predicted mortality: (y) = ℮^X^ / (1 + ℮^X^). X = − 1.853 + 1.905 × (Injury severity score > 35) + 1.625 × (Shock/CA requiring VA-ECMO)

**Table 5:** Reports focused on extracorporeal membrane oxygenation used in patients presenting traumatic brain injury

Author (Country; Year)	Therapeutic purpose	Severity of TBI	Case Number; Median age (y)/ ISS	Device /ER-to-device time	Anticoagulation/ Hemorrhagic event	Mortality/ Median Device Time
Reynolds[19] (USA/1999)	Ventilation assistance	TBI (AIS 3) ICP monitor: No	1 case; 16/18	VV-ECMO /0 (d)	Heparin free/ Pulmonary hemorrhage	No /7 (d)
Yen [20] (Taiwan/2008)	Hemodynamic assistance	TBI (AIS 5) ICP monitor: Yes	1 case; 21/NA*	VA-ECMO /2 (d)	Heparin free/ No hemorrhagic event	No /49 hours
Messing [21] (USA/2014)	Ventilation assistance	TBI (AIS ≥ 3) ICP monitor: Yes	1 case; 21/38	VV-ECMO /3 (d)	Initial heparin free then ACT 180-200 s/ No hemorrhagic event	No /20 (d)
Muellenbach [22] (Germany/2012)	Ventilation assistance	TBI (AIS ≥ 3) ICP monitor: Yes	3 cases; 53/59, 16/66, 28/66	VV-ECMO /2 (d), 0 (d), 0(d)	Initial heparin free then aPTT 40-60 s/ No hemorrhagic event	No /8 (d), 3 (d), 3(d)
Munoz-Bendix [23] (Germany/2015)	Reducing PaCO_2_ for ICP reduction	TBI (AIS >3) ICP monitor: Yes	10 cases; 50 /NA*	pECLA / 6(d)	aPTT 50-60 s/ NA*	NA* /8 (d)
Biscotti [24] (USA/2015)	Ventilation assistance	TBI (AIS >3), ICP monitor: Yes	2 cases; 18/27, 20/33	VV-ECMO /4 (d), 4(d)	aPTT 40-60 s/ No hemorrhagic event	No / 6(d), 13 (d)
Zhou [25] (China/2015)	Ventilation assistance during tracheal repair	TBI (AIS 3), ICP monitor: No	1 case; 33/34	VA-ECMO /4 (d)	ACT 300 (s)/ No hemorrhagic event	No /551 minutes
Robba [26] (UK/2017)	Ventilation assistance	TBI (AIS ≥ 3) ICP monitor: Yes	2 cases; 31/41, 54/32	VV-ECMO /5 (d), 20 (d)	NA*/ No hemorrhagic event	50% (n=1) /20 (d), 4 (d)

*ACT:* Active clotting time. *APTT:* Activated partial thromboplastin time. *ECMO*: Extracorporeal membrane oxygenation. *ICP*: Intracranial pressure monitor. *pECLA*: Pumpless extracorporeal lung assist. *TBI*: Traumatic brain injury. *VA*: Venoarterial. *VV*: Venovenous

*NA: Not available

## Discussion

This study is aimed at identifying the independent predictors of hospital mortality in adult trauma patients receiving ECMO for advanced cardiopulmonary dysfunctions. Due to the complicated technique and the high resource demand, ECMO is not a routine therapy for advanced cardiopulmonary dysfunctions. However, after experience accumulation, it can become a valuable salvage treatment for advanced cardiopulmonary dysfunctions induced by various etiologies. Based on our experience of handing the coagulopathy in patients requiring postcardiotomy ECMO support [16], we considered that all the surgical or trauma candidates must have their major bleeding sites identified and controlled before the delivery of ECMO. This is the most important selection criterion in our practice [8, 9]. Once ECMO is delivered to a patient with a high risk of hemorrhage, the heparin effect must be minimized even reversed during the support of ECMO [8, 9]. According to the current study, the mortality rates of the ECMO-treated post-traumatic shock and respiratory failure were 64% and 27%, respectively. Compare to our previous publications, the mortality rate of the ECMO-treated post-traumatic shock here was similar to the mortality rate of the ECMO-treated postcardiotomy shock (64% vs. 58%; *p* = 0.66) [16]. In the other hand, the mortality rate of the ECMO-treated post-traumatic respiratory failure here was lower than the mortality rate of the ECMO-treated non-traumatic respiratory failure (27% vs. 51%; *p* = 0.05) [17]. Therefore, we thought that ECMO could be a useful life support for advanced post-traumatic cardiopulmonary dysfunctions with a careful patient selection.

In the current study, an ISS > 35 and a refractory post-traumatic shock/CA were found to be independent predictors of hospital mortality in these ECMO-treated trauma patients. The two factors may build a simple classification model for this patient population. According to the Table 3, both the observed and predicted hospital mortality rates were over 80% in patients with the two features. A similar result is found while the classification was applied to a published cohort of 18 adult trauma patients who required ECMO support [4]. Therefore, the patient who has an ISS > 35 and developing a refractory post-traumatic shock/CA should be a suboptimal candidate for ECMO resuscitation, and the decision of enrolling should be made carefully. On the contrary, ECMO seems to be a promising therapeutic option for advanced post-traumatic respiratory failure. Post-traumatic respiratory failure is a common complication seen in patients with blunt chest trauma. Except for pulmonary contusions and transfusion-induced acute lung injury, there are some specific conditions may hinder the work of MV in trauma patients. These conditions include a significant air leak due to pulmonary lacerations, uncoordinated thoracic cage movements due to complex fractures, and restrictive intra-thoracic spaces due to abdominal compartment syndrome. Once thoracotomy is required for hemorrhage control, the associated one-lung ventilation may also bring additional volutrauma to the lungs and increase the risk of advanced respiratory failure after operation. Some of these injuries require surgical repairs but some are improved with lung rest and negative fluid balance. All these can be safely managed by an experienced ECMO team with multidisciplinary expertise [6, 8]. Therefore, if the major bleeding sites are controlled, VV-ECMO is indeed a valuable option to assist these young and previously healthy adults to conquer the temporary respiratory failure and survive.

In this study, twelve of the ECMO-treated trauma patients developed major hemorrhages on ECMO. Nine of the 12 hemorrhagic patients had a prolonged aPTT (> 40 s) before the administration of ECMO. As shown in Fig. 2, ECMO induces an abrupt rise of aPTT in all patients, including the patients treated with the heparin-minimized strategy. Patients with a prolonged aPTT before ECMO institution are quite vulnerable to the anticoagulation effects of heparin and might still lose blood from the oozing wounds due to the difficulty in formatting steady blood clots on ECMO, although the major bleeding sites had been controlled. Reversing the heparin with protamine, mitigating the consumption coagulopathy with blood transfusion, stabilizing the formed blood clots with antifibrinolytic agents, and allowing permissive hypotension are common strategies used to maintain an acceptable output of ECMO before hemostasis is achieved. However, permissive hypotension may inevitably prolong the period of hypoperfusion and jeopardize the survival rate. Therefore, the scale and duration of this hypocoagulation period should be a critical target for intervention while using ECMO to rescue patients with significant TIC. Despite not used in our practice yet, the viscoelastic assay is another valuable technique of coagulation monitoring in this scenario [3, 18].

Regardless of the indications, applying ECMO to patients with traumatic brain injury (TBI) is still a controversial issue. In the current study, four patients developed major ICHs on ECMO. One patient had a re-bleeding TBI, and 3 patients had newly developed ICHs. All of them received ECMO soon after admission, with a median ER-to-ECMO time of 11 h. In the other hand, the TBIs did not re-bleed in the other 3 patients who had a longer ER-to-ECMO time (47, 248 and 295 h, respectively). Therefore, the ER-to-ECMO time seemed to have an effect on re-bleeding from TBIs during ECMO, although a heparin-minimized strategy was adopted. Due to the small sample size, it is impossible to draw any definite conclusion to document the feasibility of delivering ECMO to patients with known TBI. To shorten this knowledge gap, we decided to perform a simple literature review of the case reports focusing on applying ECMO or similar device to patients with TBI. We collected eight English publications from PubMed that were published from 1999 to 2017 [19–26]. The eight studies, enrolling 21 patients with known TBI before ECMO, are summarized in Table 5. The therapeutic purpose is ventilation assistance in seven of the eight studies. The severity of TBI is significant in 19 patients in whom an extraventricular drainage is placed for intracranial decompression and pressure monitoring. Most of the patients have an ER-to-device time > 2 days, and a heparin-free ECMO is provided to patients with an ER-to-device time < 3 days. No TBI re-bleeding occurs, and just one hospital death is reported. According to the above-mentioned findings, ECMO with a heparin-free strategy seems to be safe in patients with minor or drained TBI. However, the summary could be more comprehensive if the therapeutic ranges of aPTT of the “heparin-free” ECMO are available.

The limitations of this study are its retrospective design and the small number of cases involved. The observations were obtained from the ECMO-treated patients only and all of the patients were highly selected by the ECMO team. Further prospective or large-scale retrospective studies are needed to demonstrate the efficacy and safety of ECMO used for post-traumatic cardiopulmonary failure.

## Conclusion

The severity of trauma and the type of cardiopulmonary dysfunction significantly affected the outcomes of ECMO used for sustaining adult patients with post-traumatic cardiopulmonary dysfunction. According to our results, ECMO could be a useful salvage therapy for adult patients with post-traumatic cardiopulmonary dysfunctions if they had an ISS less than 35. Hemorrhage on ECMO remained a true concern while the device was required soon after trauma, although a heparin-minimized protocol was adopted.

## Appendix 1

**Table 6:** Summary of major injuries in all patients

Case	ISS	Major Injury
VA1	10	Right HxTx. Scalp lacerations.
VA2	13	Right HxTx. Grade 2 hepatic injury.
VA3	41	3rd degree burn (TBSA35%). Left flail chest with open PnTx. Right femoral bone fracture.
VA4	75	Cardiac arrest. Pelvic crush injury with hemorrhagic shock. Bilateral lung contusions.
VA5	75	Cardiac arrest. Interstitial pulmonary edema.
VA6	25	2–3 degree burn (TBSA50%).
VA7	36	Left HxPnTx. Cerebral contusions with coma > 6 h. Minor pelvic fracture.
VA8	25	Blunt cardiac injury with left anterior descending artery disruption.
VA9	16	2–3 degree burn (face involved; TBSA 10%) and inhalation injury.
VA10	43	Grade 5 splenic lacerations and grade 2 renal lacerations. Right small ICH. Minor facial bone fracture.
VA11	50	Pelvic crush injury with hemorrhagic shock. Grade 4 splenic and grade 3 hepatic lacerations. Diaphragmatic rupture. Bilateral HxPnTx with lung contusions. Traumatic SAH.
VA12	20	Right HxPnTx with lung contusion. Right humeral and left tibial fractures.
VA13	38	Pelvic crush injury with hemorrhagic shock. Right HxPnTx. Right tibia fracture.
VA14	18	Right lung contusion. Mesentery vascular injury. Minor L-spine fracture.
VV1	20	Left flail chest with open PnTx. L-spine minor fracture.
VV2	17	Bilateral lung contusions. Scalp lacerations.
VV3	29	Bilateral HxPnTx with multiple ribs fracture. C-Spine minor fracture.
VV4	34	Bilateral lung contusions. Grade 3 hepatic lacerations. LeFort I facial and right femoral bone fracture.
VV5	75	Cardiac arrest. Blunt cardiac injury with large hemopericardium.
VV6	75	Cardiac arrest. Bilateral lung contusions. Grade 4 hepatic lacerations.
VV7	9	2–3 degree burn (face involved; TBSA 8%) and inhalation injury.
VV8	41	Pelvic crush injury with hemorrhagic shock. Left flail chest with lung contusion. Left femoral bone fracture.
VV9	29	Bilateral lung contusions with HxTx. Right femoral bone fracture. L-spine minor fracture.
VV10	75	Cardiac arrest. Grade 4 hepatic lacerations.
VV11	75	Cardiac arrest. Pelvic crush injury with hemorrhagic shock. Mesenteric vascular injury. Urinary bladder disruption. Multiple ribs fractures with right HxTx.
VV12	22	Right HxPnTx. Stomach and diaphragmatic perforation. Head concussion.
VV13	43	Blunt cardiac injury with cardiac rupture. Facial bone and skull base fractures with pneumocranium and SAH. Mesenteric injury. Small bowel disruption.
VV14	25	Blunt injury of urinary bladder with bladder disruption. Bilateral femoral bone fractures.
VV15	34	Bilateral flail chest with left HxPnTx. Left femoral bone fracture.
VV16	29	Bilateral HxPnTx. Right proximal femoral bone fracture. L-spine minor fracture.
VV17	45	Blunt injury of thoracic aorta with pseudoaneurysm formation. Pelvic crush injury with hemorrhagic shock. Grade 4 hepatic lacerations and mesenteric vascular injury. Minor facial bone fracture.
VV18	18	Skull base fracture. Left HxPnTx.
VV19	10	Traumatic SAH. Minor facial lacerations.
VV20	17	Left renal grade 4 lacerations. Left 11th rib fracture.
VV21	25	Bilateral HxPnTx and lung contusions. Grade 3 liver lacerations.
VV22	50	Blunt injury of thoracic aorta with pseudoaneurysm formation. Grade 5 hepatic lacerations.

*HxTx* Hemothorax, *PnTx* Pneumothorax, *HxPnTx* Hemopneumothorax, *ICH* Intracranial hemorrhage, *SAH* subarachnoid hemorrhage

## Appendix 2

**Table 7:** Patient information

NO	Age (year)	Trauma Mechanism	ISS	ER-to-ECMO hour	Pre-ECMO management		Outcome (ECMO hour)
					MV hour	Damage control surgery/TAE	
VA-ECMO for post-traumatic shock							
1	37	Stabbing	10	4	3	Thoracotomy: lung wedge resection	Survived (112)
2	48	Traffic accident (car)	13	4	13	Thoracotomy: Cardiorrhaphy	Survived (91)
3	29	Electrocution	41	6	1	No	Dead (17)
4	17	Traffic accident (car)	75	6	2	TAE for retroperitoneal hemorrhage External fixation of pelvic fracture	Dead (216)
5	25	Near-drowning	75	8	2	No	Survived (384)
6	42	Burn	25	10	6	No	Dead (36)
7	33	Falling	36	20	16	Thoracotomy: RLL and LLL lobectomy	Dead (297)
8	20	Traffic accident (car)	25	24	10	Sternotomy: CABG for LAD disruption	Survived (216)
9	61	Burn and inhalation	16	26	26	No	Dead (212)
10	35	Traffic accident (car)	43	37	36	Laparatomy: Splenectomy and pancreatic tail resection	Dead (23)
11	47	Traffic accident (car)	50	47	50	Thoracotomy: RLL Wedge resection Laparotomy: Diaphragmatic repair, splenectomy, hepatorrhaphy and retroperitoneal packing	Dead (69)
12	45	Traffic accident (car)	20	48	46	No	Dead (258)
13	40	Falling	38	48	60	TAE for retroperitoneal hemorrhage External fixation of pelvic fracture	Dead (43)
14	24	Traffic accident (motorbike)	18	315	288	Laparotomy: Small bowel resection with end-ileostomy	Survive(116)
VV-ECMO for post-traumatic respiratory failure							
1	53	Traffic accident (pedestrian)	20	5	4	No	Survived (69)
2	29	Traffic accident (motorbike)	17	7	6	No	Survived (61)
3	33	Traffic accident (motorbike)	29	9	5	Thoracotomy: RML-RLL bilobectomy, pericardiotomy	Survived (187)
4	33	Traffic accident (car)	34	10	8	No	Survived (70)
5	59	Traffic accident (car)	75	11	9	Subxiphoid drainage	Survived (75)
6	23	Falling	75	14	9	Thoracotomy: Repair RML/RLL lacerations Laparotomy: Ligation of right hepatic artery with perihepatic packing	Dead (40)
7	49	Burn and inhalation	9	16	18	No	Survived (177)
8	49	Traffic accident (pedestrian)	41	16	14	TAE for retroperitoneal hemorrhage, external fixation of pelvic fracture	Survived (222)
9	43	Traffic accident (motorbike)	29	21	4	No	Survived (77)
10	20	Traffic accident (motorbike)	75	23	21	TAE for grade 4 hepatic laceration. Laparotomy: Ligation of right hepatic artery and right portal vein with perihepatic packing	Dead (24)
11	57	Traffic accident (car)	75	52	49	TAE for retroperitoneal hemorrhage, external fixation of pelvic fracture. Laparotomy: Resect terminal ileum and right colon with end-ileostomy for bowel perforation	Dead (423)
12	37	Falling	22	76	74	Laparotomy: Repair gastric and diaphragmatic perforation, repair spleen avulsion	Survived (66)
13	61	Traffic accident (motorbike)	43	100	91	Laparotomy: Repair small bowel perforation	Survived (143)
14	27	Compression injury	25	115	40	Laparotomy: Cystorrhaphy	Survived (161)
15	25	Traffic accident (car)	34	123	108	No	Survived (287)
16	32	Traffic accident (motorbike)	29	138	126	No	Dead (1030)
17	28	Traffic accident (car)	45	175	143	TAE for grade 3 hepatic laceration Laparotomy: Repair mesocolonic laceration	Survived (94)
18	72	Traffic accident (motorbike)	18	248	227	Thoracoscopy for empyema evacuation	Dead (111)
19	56	Traffic accident (motorbike)	10	295	150	No	Survived (517)
20	58	Falling	17	334	310	TAE for grade 4 renal laceration	Survived (169)
21	25	Traffic accident (pedestrian)	25	384	365	TAE for grade 3 hepatic laceration Thoracotomy: RML-RLL bilobectomy and bronchoplasty Exploratory Laparotomy	Survived (456)
22	27	Traffic accident (motorbike)	50	574	174	TAE for grade 5 hepatic laceration Laparoscopy for persistent bile leak and intra-abdominal abscess Thoracotomy: Descending aortic replacement	Dead (352)

*CABG* Coronary artery bypass grafting, *CA* Cardiac arrest, *ECMO* Extracorporeal membrane oxygenation, *ER* Emergency room. *ISS* Injury severity score, *LAD* Left anterior descending, *MV hour* Mechanical ventilation hours before ECMO, *TAE* Transcatheter arterial embolization, *RML* Right middle lobe of lung, *RLL*: Right lower lobe of lung

## Abbreviations

APTT: Activated partial thromboplastin time
CA: Cardiac arrest
CRRT: Continuous renal replacement therapy
ECMO: Extracorporeal membrane oxygenation
ER: Emergency room
FiO2: Fraction of inspiratory oxygen
ICH: Intracranial hemorrhage
ISS: Injury severity score
MV: Mechanical ventilation
PaCO_2_: Arterial carbon dioxide tension
PaO_2_: Arterial oxygen tension
ROC: Receiver operating characteristic
SaO_2_: Arterial oxygen saturation
SpO_2_: Oxyhemoglobin saturation by pulse oximetry
TBI: Traumatic brain injury
TIC: Trauma induced coagulopathy
VA: Venoarterial
VV: Venovenous
